# Molecular Biology for Diagnosis of Congenital and Neonatal Infections in the Cerebrospinal Fluid of Newborns from a Brazilian Tertiary Hospital

**DOI:** 10.3390/microorganisms12112133

**Published:** 2024-10-24

**Authors:** Suzana Ferreira Zimmerman, Rodrigo Gonçalves de Lima, André Moreno Morcillo, Sandra Helena Alves Bonon, Sergio Tadeu Martins Marba

**Affiliations:** 1 Department of Pediatrics, School of Medical Sciences, FCM Unicamp, Campinas 13083-970, SP, Brazil; 2 Virology Laboratory, School of Medical Sciences, FCM Unicamp, Campinas 13083-970, SP, Brazil

**Keywords:** newborn, congenital Toxoplasmosis, Cytomegalovirus infections, Zika virus infection, polymerase chain reaction, cerebrospinal fluid

## Abstract

The risk of infection transmission from mother to fetus depends on the pathogen. TORCH agents cause some neuroinfections, including Toxoplasmosis, rubella, Cytomegalovirus, herpes simplex 1 and 2, and others (Varicella Zoster, Parvovirus B-19, Epstein–Barr virus, and Zika virus). The consequences can be stillbirth, prematurity, uterine growth restriction, and congenital malformations. The detection of DNA/RNA from CSF by molecular methods is a marker of the involvement of congenital infection in the central nervous system. This study aimed to identify the frequency of these pathogens in CSF samples from newborns (1 to 28 days old) at a tertiary hospital, using PCR, and determine the clinical consequences. Methods: This was a prospective descriptive study involving the molecular analysis of 151 CSF samples from neonates, collected for cytological and biochemical diagnosis from 2017 to 2021. After the results and consent from the participants’ caregivers were obtained, the leftover material was sent to the University’s Virology Laboratory and submitted for DNA/RNA extraction and Nested-PCR/RT-PCR. A review of the patients’ medical records and descriptive statistics was performed. This work was approved by the Ethics Committee (CAAE: 86760218.3.0000.5404). Results: A total of 151 CSF samples were obtained, 16 of which were positive (10.6% [95% CI%: 6.18–16.63%]). Two of these were PCR-positive for HSV-1 (1.3%), four for VZV (2.6%), one for CMV (0.67%), two for Toxoplasmosis (1.3%), four for Parvovirus B-19 (2.6%), and four for Zika (2.6%). The proportion of positive PCR results was higher in the group that presented with malformations (25.0% vs. 8.4%, *p* = 0.040). Conclusions: The pathogens identified by PCR were mostly Zika virus, VZV, and B-19, and these were mainly found in newborns with malformations.

## 1. Introduction

Infections caused by viruses, bacteria, and protozoa can be transmitted from mother to fetus or newborn and cause signs and symptoms in them. Diseases can be transmitted in the prenatal period through the hematogenous and transplacental route or at the time of delivery and postnatally through contact with blood or vaginal secretions [1].

The risk of transmission from mother to fetus depends on etiological agents and the gestational trimester. Usually, the most severe conditions are acquired during the first trimester. As a consequence of this infectious process, embryo resorption, abortion, stillbirth, congenital malformations, prematurity, intrauterine growth restriction (IUGR), acute disease of the newborn (NB) in the uterus or postpartum, or persistent asymptomatic infection in the neonatal period with neurological sequelae can occur [2].

Some of these infections come from TORCH pathogens: Toxoplasmosis, rubella, Cytomegalovirus (CMV), herpes simplex types 1 and 2 (HSV-1 and 2), and others (such as Varicella Zoster (VZV), Epstein–Barr virus (EBV), Parvovirus B-19, Zika virus, and syphilis). The acronym TORCH is well known in the neonatal/perinatal medicine field, and since 2016, the addition of the letter Z (TORCHZ), referencing congenital Zika virus infection, has been suggested [3]. The detection of DNA/RNA in cerebrospinal fluid (CSF) by the polymerase chain reaction (PCR) is a marker of congenital infection in the nervous system [2].

In this study, we aimed to identify the primary pathogens causing congenital infection in CSF samples from newborns (NBs), seen at a Brazilian tertiary hospital, through the use of a molecular methodology. In addition, we aimed to identify the clinical picture and congenital malformations present in patients carrying TORCHZ’s pathogens by evaluating the medical records of newborns suspected of congenital infection from that tertiary hospital.

## 2. Methods

This study was prospective descriptive in nature. CSF samples were obtained from neonates (1 to 28 days old) with suspected congenital infection, cared for at the Neonatal Unit of a tertiary hospital, from 2017 to 2021. The samples were sent for cytological and biochemical research, and a request for the clinical conduction of the case was sent to the Laboratory of Biological Fluids of the Department of Clinical Pathology of a Brazilian tertiary hospital. The remaining material was stored in a freezer at −20 degrees Celsius and sent to the School of Medical Sciences’ Virology Laboratory, where it was stored in a freezer at −80 °C.

No additional collections were requested for the study, causing no harm to patients. This work was approved by the Ethics Committee on Research in Human Beings, following the standards outlined in resolution Nº466/12 of the National Health Council. It was approved via Plataforma Brasil (CAAE: 86760218.3.0000.5404) on 5 June 2018.

Once thawed, the CSF samples were subjected to DNA and RNA extraction and used for molecular analysis of the presence of the studied pathogens’ genomes; the samples were analyzed for all pathogens mentioned. Next, PCR and Nested-PCR (NPCR) were performed, and for the Zika virus, a real-time PCR (RT-qPCR) was conducted.

The sample’s DNA was extracted from 200 µL of CSF using BIOPUR’s Mini Spin Plus kit-250, Mobius Life Science Company, located in Pinhais, Paraná, Brazil. The manufacturer’s guidelines were followed. To monitor the presence of inhibitors, Beta2-microglobulin gene primers (F: GGTGTCTTGAGGCTCAGGGAG; R: CAACTTCAATGTCAATGTCGATGGATG) were included in each sample as a control. DNA extraction was repeated when the PCR samples for the Beta2-microglobulin gene were not positive. The samples’ RNA was extracted from 140 µL of the CSF sample using the SV Total RNA Isolation System. “Bio Gene Extração de DNA/RNA Viral” kit, Quibasa Company, located in Belo Horizonte, Minas Gerais, Brazil. The Access Quick^®^ RT-PCR System kit (“High-Capacity cDNA Reverse Transcription Kit”, 200 reactions, Applied Biosystems by Thermo Fisher Scientific, Ref 4368814, manufactured in Vilnius, Lithuania) was used for the reverse transcription (RT) reaction. Then, the cDNA was obtained and stored at −20 °C.

PCR and NPCR were performed with a total volume of 10 µL, containing 0.5 µL of DNA extracted for PCR (and 0.5 µL of PCR product for NPCR), 5.0 mL of GoTaq^®^ Green G2 Master Mix M7823 (Promega, Winchester, VA, USA), 0.5 µL of each primer, and 3.5 µL of ultrapure water. The electrophoretic analysis was performed with 5.0 µL of the amplified material of the NPCR in 2% agarose gel, stained with Unisafe Dye (Uniscience, Osasco, Brazil), and subjected to electrophoresis under ultraviolet light to visualize the DNA/RNA bands of the specific amplicons of each pathogen. 

Real-time PCR was performed using TaqMan™ Fast Advanced Master Mix (Applied Biosystem, Waltham, MA, USA), by Thermo Fisher Scientific, Ref 4444557, manufactured in Vilnius, Lithuania/Waltham, MA, USA. The primers and hydrolysis probes (sequences already described in the literature) synthesized as PrimeTime qPCR Primers (Integrated DNA Technologies Inc., Coralville, IA, USA) were also used. The tests were performed in the System (Applied Biosystem StepOnePlus ™ Real-Time PCR): 95 °C by 20 s, 40 cycles of 95 °C at 1 s, and 60 °C for 20 s.

The variables of the newborns and mothers collected from the medical records were the following: gestational history of mother, neonatal sex, age, weight, head circumference and length at birth; gestational age, Apgar score at 1st and 5th min, anomalies of any system, presence or absence of microcephaly and other complications, with corresponding complementary exams and clinical evolution: initial diagnostic hypothesis, cytological and biochemical results of CSF exams (records of proteins, glucose, red blood cells, and leukocytes; in addition to bacterioscopy and culture of CSF samples). One of the initial clinical diagnostic hypotheses was sepsis, currently defined as follows: syndrome, in the first month of life, with clinical and laboratory signs of infection, often accompanied by bacteremia. Another diagnosis was the following: exposure to syphilis during pregnancy—this is what happens when a pregnant person is infected with the bacterium *Treponema pallidum* and can transmit the infection to their fetus. 

This information was described using absolute values (n), prevalences, percentages, and descriptive statistical results, for example, median and expected values.

The variables were studied descriptively in the statistical analysis using SPSS 16.0 software (SPSS Inc., Chicago, IL, USA) and Stata 12.0 (StataCorp, College Station, TX, USA). Categorical variables are presented in tables containing absolute and relative frequencies. The mean, median, standard deviation, quartiles, minimum and maximum values, and maximum and interquartile amplitudes were determined for the quantitative variables. The Mann–Whitney test was used to compare age distributions regarding sex. The percentage of positive tests for each reaction was determined—with the respective 95% confidence intervals—by the exact method using binomial distribution. Fisher’s exact test or the Fisher–Freeman–Halton exact test was used to evaluate the association between two categorical variables. A significance level of 5% was adopted for all analyses.

## 3. Results

First, 151 CSF samples were obtained from the newborns of a tertiary hospital to investigate the presence of TORCHZ pathogens. The median age of the NB included in this study was three days, and the average was 6.41 days. Females predominated (53.3%)—Table 1. There was no statistically significant difference in the distribution of age regarding sex.

The most frequent causes of CSF collection were: sepsis (61 samples = 40.4% of the total); congenital malformation (20 = 13.2%), including microcephaly (3 samples = 2.0%); seizures (19 = 12.6%); exposure to syphilis during pregnancy (42 = 27.8%); others, such as respiratory distress, febrile exanthematous disease during pregnancy, neonatal death, birth parent with CMV, moderate hypoxic–ischemic encephalopathy, intrauterine growth restriction, hypertonic syndrome, hyperexcitability syndrome, gastroschisis, minor dysmorphisms, and small caudate nucleus head cyst—Table 1.

Among these 151 CSF samples from the tertiary hospital’s neonates, positive results were obtained in 16 of all the evaluated samples (10.6% detection of pathogen DNA or RNA in cerebrospinal fluid [95% CI: 6.18–16.63%]. Out of the 16 samples, 15 were positive for only one PCR reaction, and 1 was positive for two PCR reactions (VZV and Parvovirus B-19). Two of these samples had positive PCR for HSV-1 (1.3%), four samples were positive for VZV (2.6%), one was positive for CMV (0.7%), two were positive for Toxoplasmosis (1.3%), four were positive for Parvovirus B-19 (2.6%), and four were positive for Zika virus (2.6%)—the frequencies of positive PCRs for HSV1, VZV, HCMV, TOXO, B-19, and Zika are presented in Table 2.

There was no association between positive PCR and diagnostic groups in the different agents (for each pathogen alone). This means that it cannot be said that there is a statistically significant difference between the percentage of positive PCR for each pathogen individually between the diagnostic groups. The PCR reactions of HSV-2 and EBV were all negative.

Among the malformations mentioned in the medical records of patients whose CSF had been collected, we found the following: two patients with Arnold Chiari Type II malformation and another with occipital encephalocele and microcephaly (one of each positive for Parvovirus B-19); nine cases of hydrocephalus (one positive for Zika virus); one case of lumbosacral myelomeningocele (positive for Zika); one twin was found to have occipitocervical cyst, dysraphism, and ventriculomegaly, while his brother had occipital encephalocele; two patients with macrocrania; three cases of microcephaly (one positive for Toxoplasmosis and one for Parvovirus B-19).

The global evaluation of PCR reactions regarding the diagnostic groups is presented in Table 3. The proportion of positive PCR (for all pathogens) was higher in the group that showed malformations [25.0% vs. 8.4%]—statistically significant difference: *p* = 0.040, that is, *p* < 0.05. When this relation of positive PCR was evaluated with other diagnoses individually, there was no difference in the percentage of positive PCR and seizure, sepsis, or syphilis exposure, nor with other causes (respiratory distress, febrile exanthematous disease in pregnancy, neonatal death, mother with CMV+, moderate hypoxic–ischemic encephalopathy, gastroschisis, minor dysmorphisms, intrauterine growth restriction, hypertonic syndrome, hyperexcitability syndrome, small caudate nucleus head cyst)—Table 3.

In the cytology and biochemistry analyses in CSF, hyperproteinorachia > 145 mg/dL was considered. It was not possible to perform glycorrhachia analysis regarding glycemia, because it would depend on serum glucose values at the time of CSF puncture, which we did not have access to in most medical records.

Pleocytosis was considered when leukocytes > 30 cells/mm^3^. In cases of CSF with red blood cells, probably as a result of traumatic puncture, we discounted the following: a proportion of 1 leukocyte for every 500 red blood cells; in proteinorachia, 1 mg for every 500 red blood cells.

When crossing protein in CSF according to the assessment of PCR reactions, there were no statistically significant differences (Table 4) in positive PCR and hyperproteinorachia—neither globally nor individually for each pathogen.

Hyperproteinorachia analysis was performed also regarding diagnoses: there was also no difference in the percentage of hyperproteinorachia regarding diagnosis (exposure to syphilis during pregnancy, sepsis, seizure, malformation, or other causes).

When crossing between cellularity in CSF and positive PCR, globally and individually for each pathogen, there was no difference in the percentage of positive PCR and pleocytosis (Table 4).

The relation between CSF cellularity and each diagnostic group was also analyzed. There was no difference in the percentage of pleocytosis regarding each diagnostic group: malformation, sepsis, seizure, syphilis exposure during pregnancy, or other causes.

Another analysis was made by crossing between protein (biochemistry) with cellularity (cytology) in CSF: there was no association between proteinorachia and pleocytosis (*p* = 0.505: *p*—Fisher’s exact test value).

Regarding the samples included in the study, we could not verify positive PCR for syphilis’ etiological agent (*Treponema pallidum),* as we could not obtain positive control for this pathogen.

## 4. Discussion

Intrauterine infection may be suspected, sometimes, in newborns based on laboratory results obtained during pregnancy, such as positive serology for Toxoplasmosis. In the absence of suggestive maternal laboratory results, intrauterine infection may be suspected in newborns with specific clinical manifestations, including fetal microcephaly [3,4], seizures, hearing loss, rash [5], fetal dropsy, congenital heart disease, hepatosplenomegaly, jaundice, and thrombocytopenia [6].

The original concept of TORCH infections consisted of infections with similar presentations: Toxoplasmosis, rubella, Cytomegalovirus, and herpes simplex. However, others are described, such as Parvovirus B-19, Varicella Zoster, syphilis, and, more recently, Zika virus [3,4].

Furthermore, these findings are not restricted to TORCHZ infections. Some of the above signs may occur in diseases and conditions other than intrauterine infection (for example, inborn errors of metabolism or Rh blood group mismatch). Thus, the entire clinic, including maternal history and exposures, should be considered when evaluating a child for congenital infection [6].

However, sometimes it is impossible to define the diagnosis of congenital infections based only on signs and symptoms in pregnant women and even by maternal serological results, as infection may not have generated an immune response in the body.

Thus, using molecular methods to diagnose the congenital and neonatal infections investigated in this study in newborn patients undergoing cerebrospinal fluid (CSF) collection was appropriate, as it offered an overview of the most frequent infectious diseases found in the neonates included in this evaluation.

According to a systematic review [6] of molecular methods used to detect pathogen genomes in cerebrospinal fluid, 66% of encephalitis cases are caused by viruses and 26% of newborns who present with seizures have a neuroinfection of viral etiology. 

Lumbar puncture (LP) for the study of CSF in newborns is a standard procedure, as it is part of the investigation into neonates with sepsis to verify the existence of associated meningitis [7]. LP facilitates diagnosis and reduces deaths among newborns, especially those admitted to intensive care units [8].

We obtained two positive samples for Toxoplasmosis (1.3%): one patient had microcephaly of unknown cause (until this N-PCR result was obtained), and another was exposed to syphilis during pregnancy, which motivated CSF collection.

Toxoplasmosis is a disease caused by the protozoan *Toxoplasma gondii* with wide geographic distribution and a high serological prevalence of 60% [6]. Congenital Toxoplasmosis occurs when a pregnant person acquires an acute infection during pregnancy; the consequences of this for the fetus will depend on factors such as the degree of exposure of the fetus and the period of gestation [6]. Congenital Toxoplasmosis is one of the most severe forms of the disease, which generally causes symptoms framed within Sabin’s Tetrad, in which the fetus has chorioretinitis (90% of cases), brain calcifications (69%), neuropsychomotor development restriction (60%), and cranial volume changes with macro- or microcephaly (50% of cases) [6,9], as presented by one of the positive patients in our sample.

In one study performed by Olariu et al. [10], the PCR was positive in CSF, with sensitivity in the neonates’ group of 60%. It was concluded that, in newborns whose birthing parent are not treated for Toxoplasmosis during pregnancy, CSF PCR can contribute to the early confirmation of the diagnosis of congenital Toxoplasmosis, especially in those with clinical signs [10].

Herpes simplex types 1 and 2: two HSV-1-positive samples were obtained. The reason for CSF collection was exposure to syphilis in pregnancy in both HSV-1-positive samples.

However, there was no detection of NB with HSV-2 in CSF, which is inconsistent with the literature [9,10], which describes vertical transmission from contamination with infected maternal secretion in the vaginal canal as the most frequent (HSV-2 is defined as more prevalent than HSV-1 in the CSF of newborns) [10,11,12,13,14,15,16,17,18].

Vertical transmission of herpes simplex virus (HSV) usually occurs during childbirth due to contact with infectious secretion through the vaginal canal. However, it can happen by the transplacental and postnatal routes as well [11]. This predominant mode of transmission in the birth canal should explain why HSV-2 predominates over HSV-1 in newborn CSF in the literature [6]. HSV-1 and 2 can cause different morbidities and establish latent infections that can be further reactivated, causing lesions located at the primary site of infection or close to it [6] Herpetic encephalitis can have a devastating course, with a poor prognosis. Approximately 70% of untreated patients die. Meningitis occurs in 10% of primary HSV-2 infection cases.

About 30% of neonates have HSV infection in the central nervous system (neurotropism), either by hematogenous dissemination or neuronal transmission [12,13], but the symptoms are usually nonspecific [14,15,16,17,18].

Four samples had positive PCRs for Varicella Zoster (VZV). The initial reason for collection was infectious screening in 50% of cases, one for respiratory distress and the other for exposure to syphilis in pregnancy. Congenital Varicella syndrome can be characterized by an embryofetopathy, which includes cicatricial skin lesions, limb hypoplasia, muscle atrophy, clubfoot, intrauterine growth restriction, microcephaly, cerebellar and cortical atrophy, hydrocephalus, seizures, and intracranial and extracranial calcifications [6]. In case of neurological involvement, newborns can present with cerebellar ataxia, acute myelitis, optic neuritis, polyradiculoneuritis, CSF with an eventual increase in lymphocytic cellularity, meningoencephalitis, and seizures [6].

The literature has documented vertical transmission of Epstein–Barr virus. Although it is rare, it can occur in the first trimester of gestation: infection of the fetus can lead to a syndrome with various congenital anomalies [19] (micrognathia, cryptorchidism, cataracts, hypotonia, thrombocytopenia, persistent monocytosis, proteinuria, and metaphysitis at birth) [6]. However, all CSF samples from neonates included in this study had negative PCR results for this pathogen.

Although the literature cites Cytomegalovirus (CMV) as the most frequent cause of congenital infection [19,20,21,22,23], this was not found in this sampling: only one CSF sample with positive PCR was taken from a patient with suspected meningitis. According to a systematic review of molecular detection of congenital and neonatal infections caused by TORCH [6], CMV congenital infection is asymptomatic in most full-term newborns. However, it may be associated with severe clinical conditions, such as the “Sepsis-like” syndrome, cholestasis, thrombocytopenia, neutropenia, and pneumonitis, when affecting preterm newborns with birth weight minor to 1.500 g or a gestational age of less than 32 weeks [6].

Positive PCR in CSF of neonates with clinical symptoms of CNS infection at birth is described as the most common case [20]. Congenital CMV infection is a significant public health problem due to the high risk of late adverse consequences in both symptomatic and asymptomatic children at birth. Approximately 0.5% to 1% of newborns are estimated to be infected with CMV due to congenital infection [20,21,22].

Symptomatic newborns with CMV neuroinfection at birth usually have a poor prognosis. About 90% may evolve with neurological sequelae and 50% to 70% may evolve with bilateral and profound sensorineural deafness [23]. This happened to the neonate with congenital CMV included in this study. The involvement of the CNS must be evaluated with special attention in the presence of sensorineural deafness and neuropsychomotor development delay [23]. Lethality in symptomatic newborns with severe systemic involvement in the neonatal period can range from 5% to 10% [24].

Four samples had DNA detection of Parvovirus B-19 in the CSF of neonates: two with suspected sepsis and two with congenital malformation in the CNS—one of them had occipital encephalocele and microcephaly, another had Arnold Chiari Malformation Type II/Lumbar spina bifida with hydrocephalus.

Vertical transmission occurs in 30% of cases of maternal infection, more frequently in the first and second trimesters of pregnancy, which can cause dropsy and fetal death, due to a drop in the number of erythrocyte precursors causing intense anemia. Laboratory tests are needed mainly when the birthing parent presents with a rash [24]. However, there are cases of asymptomatic infections that do not exclude transmission to the fetus [24].

Four samples showed the presence of Zika virus RNA in the CSF of NB, detected through real-time PCR. One of these patients had spina bifida/corrected route lumbosacral myelomeningocele/Arnold Chiari Syndrome Type 2 and hydrocephalus. Another patient had only hydrocephalus, while two others were undergoing sepsis investigation/infectious screening. In April 2015, the spread of Brazil’s Zika virus (ZIKV) was confirmed as an etiological agent causing acute exanthematous disease [3,4]. From October of the same year, a microcephaly epidemic was related to a maternal infection by ZIKV during the gestational period, with indications of congenital transmission. Other causes of congenital infection with similar manifestations, such as Cytomegalovirus and Toxoplasmosis, as well as other genetic or environmental causes, were ruled out [3,4]. Other signs of this congenital infection (calcifications, ventriculomegaly, and cortical development disorder) comprised the congenital Zika Syndrome, an epidemic of which was established in 2015. Moreover, 2205 cases were confirmed by the end of 2016 [6].

The World Health Organization (WHO) defined microcephaly as a head circumference (CP) equal to or less than 31.9 cm for male infants and equal to or less than 31.5 cm for female infants born at term [3,4]. The most likely cause of this decrease is related to the presence of the virus in the CNS, promoting neuronal death directly or by activation of immune responses of infected hosts, compromising the structure and functioning of important areas [25].

In their case–control studies, de Araujo et al. [3,4] included cases that were newborns with microcephaly and controls that were neonates without microcephaly. Birthing parents of newborns with microcephaly (cases) had more serological markers of previous ZIKV infection compared to the birthing parents of controls (without microcephaly), although birthing parents in both groups were PCR-negative [3,4]. Among newborns, 33 of 91 cases (35%) and 173 control infants had no laboratory-confirmed ZIKV infection. Among the 23 neonates that tested positive for ZIKV, there were 10 that had brain abnormalities (43%) and 13 that had no brain abnormalities, while 11 of 56 (20%), that were laboratory-confirmed negative for ZIKV, had brain abnormalities. The association between microcephaly and congenital ZIKV infection has been confirmed [3,4].

Pomar et al. [25] studied maternal–fetal transmission and it seemed to have occurred in approximately 25% of exposed fetuses. It was associated with adverse fetal and early neonatal outcomes in 33.3% of infected fetuses: fetal loss or severe signs of congenital Zika Syndrome.

The main benefit of molecular biology is to reduce the detection time of an infectious agent by avoiding the immunological window period, the time it takes for the body to create an immune response to an infectious agent. Thus, it becomes more effective compared to immunological methods that depend on the binding between antigen and antibody [26]. Molecular methods allow laboratories to quickly detect and identify earlier a pathogen earlier on that is difficult to grow in culture or those that are present in low quantities in the clinical sample [26], but they are expensive, and they are not widely distributed to routine laboratories, because of the complexity of performing them.

This study’s limitation was the impossibility of performing PCR for syphilis in the included samples, given that we did not obtain a positive control. However, it is worth noting that, in the literature, this pathogen does not have good detection sensitivity in CSF by PCR [27,28,29]. The exposure to syphilis in pregnancy already has a well-established investigation flowchart based on serological, treponemal, and non-treponemal tests [6,27,28,29], which are used in clinical practice for the increasing prevalence of this pathology in pregnant people. In this disease, the PCR was limited to research centers [6]. There are three studies with CSF analysis identifying *Treponema pallidum* by molecular tests [27,28,29]. Marangoni’s articles [27,28] described presumptive cases of congenital syphilis with positive Western Blot IgM results. From these, only some had CSF with positive PCR for Treponema. This information confirmed that prenatal syphilis serology facilitates treatment during pregnancy and reduces the risk of mother-to-child transmission. In addition, using IgM Western Blot and careful examination of CSF enabled the identification and treatment of high-risk newborns. In these studies, PCR was less sensitive because it detected fewer cases than serologic tests [27,28]. In Michelow’s study, only 35% of these children had positive CSF syphilis identified by PCR [29]. On the other hand, 13 out of 14 children with positive PCR for syphilis in CSF (93%) had positive serum IgM immunoblotting results. This study concluded that *congenital syphilis* infection can be identified in most children by physical examination, standard laboratory tests, and radiographic studies [29].

Although cerebrospinal fluid samples provide important information for diagnosing neurological infections and it is also an important part of sepsis’ investigation in newborns, the lumbar puncture is an invasive procedure that must be well indicated [6,8,30]. It is not always recommended, as described in the review conducted by the European Congenital Infection Initiative (ECCI) [31], concerning the management of congenital Cytomegalovirus infection.

Investigating vertically transmitted infectious diseases during pregnancy is critical and often the only possible way to confirm that an asymptomatic NB may be infected. However, except for Toxoplasmosis, rubella, and syphilis, the universal screening of several of the pathogens studied here that are responsible for a large contingent of cases of congenital infection, such as Cytomegalovirus, herpes simplex types 1 and 2, Varicella Zoster, Epstein–Barr virus, Parvovirus B-19, and Zika virus is not yet recommended.

## 5. Conclusions

In conclusion, the most frequent pathogens causing congenital infection in CSF samples of newborns in the Brazilian tertiary hospital studied were Zika virus, Varicella Zoster virus, and Parvovirus B-19, obtained by detecting DNA/RNA using molecular methods. The proportion of positive PCR was higher in the group that presented with congenital malformations (statistically significant difference: *p* = 0.040). This suggests the clinical relevance of investigating these pathogens using molecular methodology, for diagnosis of sepsis in newborns, and for the etiological diagnosis of congenital malformations.

## Figures and Tables

**Table 1:** Characteristics of newborns from the Neonatal Unit of a Brazilian’s tertiary hospital, with CSF samples included in the study.

Sex	N	%
Female Male	80 71	53.3 46.7
**Gestational Age**	Preterm NB (%) GA < thirty-seven weeks	Full-term NB (%) GA = from thirty-seven to forty-two weeks
	40.6	59.4
**Average age (days)**	**Median age (days)**	**Weight < 2.5 kg at birth (%)**
6.44	3	42.7%
**Initial diagnostic hypothesis that motivated CSF collection**	**N**	%
Sepsis Congenital malformations Exposure to syphilis in pregnancy Seizure Other	61 20 42 19 9	40.4 13.2 27.8 12.6 6

Total of CSF samples from CAISM neonates with congenital infection screening: 151. CSF: cerebrospinal fluid; GA: gestational age; kg: kilograms; NB: newborns. Others include febrile exanthematous disease in pregnancy; intrauterine growth restriction; moderate bilateral subdural parietal collection—secondary to cerebral atrophy—moderate hypoxic–ischemic encephalopathy; respiratory distress.

**Table 2:** Percentage of positivity of PCRs—pathogens detected in the respective CSF samples of the NB and 95% confidence interval.

Pathogens	Positive PCR	Negative PCR	% Positive PCR	CI 95% *
**TOXO**	2	149	1.3	0.16–4.70
**HSV-1**	2	149	1.3	0.16–4.70
**HSV-2**	0	151	0	0–2.41
**VZV**	4	147	2.6	0.72–6.64
**CMV**	1	150	0.7	0.02–3.63
**EBV**	0	151	0	0–2.41
**B-19**	4	147	2.6	0.72–6.64
**ZIKA**	4	147	2.6	0.72–6.64

N: total = 151 samples; HSV-1: herpes simplex type 1; VZV: Varicella Zoster; EBV: Epstein–Barr virus; CMV: Cytomegalovirus; TOXO: Toxoplasmosis; B-19: Parvovirus B-19; ZIKA: Zika virus; * CI 95%: Exact 95% confidence interval were determined by Binomial distribution; CSF: cerebrospinal fluid; NB: newborns; PCR: polymerase chain reaction.

**Table 3:** Global Evaluation of PCR Reactions regarding Patients’ Diagnosis.

Diagnosis		PCR		Total	
		positive	negative		
Congenital Malformations	Yes	5 (25%)	15	20	*p*= 0.040
	No	11 (8.4%)	120	131	
	Total	16	135	151	
Sepsis		PCR		Total	*p* = 0.306
		positive	negative		
	Yes	5	56	61	
	No	11	79	90	
	Total	16	135	151	
Seizure		PCR		Total	*p* = 0.102
		positive	negative		
	Yes	0	19	19	
	No	16	116	132	
	Total	16	135	151	
Exposure to syphilis in pregnancy		PCR		Total	*p* = 0.474
		positive	negative		
	Yes	5	37	42	
	No	11	98	109	
	Total	16	135	151	
Others		PCR		Total	*p* = 0.645
		positive	negative		
	Yes	1	8	9	
	No	15	127		
	Total	16	135	151	

N: total = 151 samples; *p* = *p*-value of one-sided Fisher’s exact test; PCR: polymerase chain reaction. Malformations: Arnold Chiari Type II, occipital encephalocele, microcephaly, hydrocephalus, lumbosacral myelomeningocele, occipitocervical cyst, macrocrania.

**Table 4:** Global evaluation of PCR and diagnostics regarding CSF’s protein and cellularity.

CSF Characteristics		PCR			*p* Value
		Positive	Negative	Total	
Pleocytosis	Yes	9	68	77	*p* = 0.423
	No	6	59	65	
	Total	15	127	142	
			PCR		
		positive	negative	Total	
Hyperproteinorachia	Yes	13	116	129	
	No	2	10	12	*p* = 0.373
Diagnostics Hyperproteinorachia	Sepsis	Malformation	Seizures	Exposure to Syphilis	Others
	49 (90.7%)	15 (78.9%)	19 (100%)	40 (95.2%)	6 (85.7%) *p* = 0.12
Pleocytosis	32 (58.2%)	11 (57.9%)	7 (36.8%)	24 (57.1%)	3 (42.9%)
					*p* = 0.528

CSF: cerebrospinal fluid; N: total = 151 samples; *p* = *p*-value of one-sided Fisher’s exact test; PCR: polymerase chain reaction.

## Data Availability

Data are contained within the article.
